# Life Cycle Assessment in the Monitoring, Reporting, and Verification of Land-Based Carbon Dioxide Removal: Gaps and Opportunities

**DOI:** 10.1021/acs.est.4c09510

**Published:** 2025-03-27

**Authors:** Yuan Yao, Bingquan Zhang

**Affiliations:** † Center for Industrial Ecology, Yale School of the Environment, 5755 Yale University, New Haven, Connecticut 06511, United States; ‡ Chemical and Environmental Engineering, Yale School of Engineering and Applied Science, 5755 Yale University, New Haven, Connecticut 06511, United States

**Keywords:** carbon dioxide removal, greenhouse gas, carbon accounting, life cycle assessment, voluntary carbon market, MRV (monitoring, reporting, and verification), carbon credits, carbon offsets

## Abstract

Life cycle assessment (LCA) has been widely used to evaluate the carbon negativity and environmental impacts of carbon dioxide removal (CDR) pathways. Various monitoring, reporting, and verification (MRV) protocols have been developed to assess the carbon credits of CDR projects within voluntary and compliant carbon markets. Many MRV protocols incorporate life cycle thinking, LCA methods, and data. This perspective examined recent LCA studies and MRV protocols published by main carbon registries, focusing on four critical land-based CDR methods: bioenergy combined with carbon capture and storage, biochar, enhanced rock weathering, and afforestation and reforestation. We compared the carbon accounting and environmental impact assessment methods employed in these LCA studies and MRV protocols to identify their methodological similarities and differences. Our analysis reveals that the LCA community can support MRV protocols by providing critical insights into baselines, additionality, uncertainty, multifunctionality, environmental safeguards, holistic emission factors, and overlooked carbon pools. We recommend that future LCA research prioritize timing, permanence, scaling, and dynamic modeling for CDR. Addressing co-benefit and land use change impact assessment will further benefit both LCA and MRV development. Collaboration between the LCA and CDR communities is essential for developing robust frameworks to support carbon markets and policymaking.

## Graphical abstract

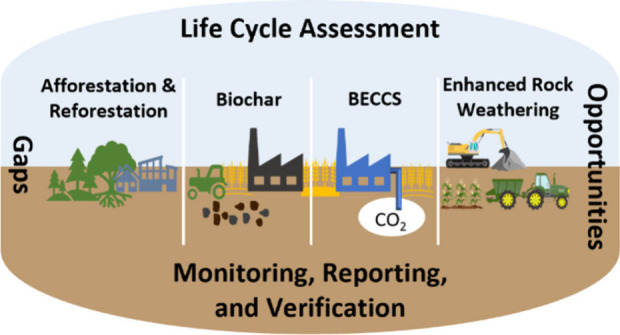

## Introduction

Carbon dioxide removal (CDR) refers to anthropogenic approaches designed to remove and durably store carbon dioxide from the atmosphere, serving as negative emission technologies. The IPCC (Intergovernmental Panel on Climate Change) emphasizes their importance in offsetting residual emissions for hard-to-decarbonize sectors and achieving net negative carbon dioxide (CO_2_) emissions.^1^ CDR methods range from engineering approaches like direct air capture and storage to nature-based solutions such as afforestation and reforestation. Many CDR strategies are land-based and can be enhanced by engineered components, forming hybrid nature-engineering CDR, such as biochar, bioenergy with carbon capture and storage (BECCS), and enhanced rock weathering. The greenhouse gas (GHG) mitigation efficacy of CDR strategies has been extensively studied.^2^ Land-based CDRs have significant potential in mitigating GHG emissions but face substantial uncertainties due to the dynamic nature of terrestrial ecosystems.^3,4^

Life cycle assessment (LCA) is a standardized and widely used approach for evaluating the environmental impacts of a product or service throughout its life cycle. Previous studies have applied LCA to CDR strategies primarily at the process (e.g., 1 biorefinery) or product level (e.g., 1 kg of biochar). Some studies have reviewed LCAs of CDR. For example, Terlouw et al. reviewed common CDR technologies, including afforestation and reforestation, biochar, soil carbon sequestration, enhanced rock weathering, ocean fertilization, and BECCS.^4^ They identified critical issues such as misinterpretation of negative emissions and the lack of consideration of temporal effects and non-climate environmental impacts. Reviews of individual CDR methods offer insights into the unique challenges associated with each method. For example, LCA reviews of BECCS indicate that land use change and ecosystem impacts are often overlooked but crucial in determining whether a BECCS project is net carbon negative.^4–6^ While biochar LCAs consistently demonstrate carbon benefits, discrepancies remain in modeling assumptions and methodological choices.^7^

Previous CDR reviews have not examined LCA in the context of MRV (monitoring, reporting, and verification). MRV involves measuring, quantifying, and monitoring CO_2_ removal of a CDR project, as well as accounting and reporting these removals.^2^ Robust MRV processes are crucial for scaling up CDR and ensuring credibility and transparency in carbon accounting across voluntary and compliant carbon markets, regulations, and national reporting. Various protocols have been developed to standardize MRV. Some MRV protocols incorporate life cycle thinking and LCA methodologies and data. A recent study proposed a carbon accounting framework for CDR that diverges from the traditional LCA approach.^8^ This framework focuses solely on quantifying carbon removal, explicitly excluding avoided emissions from the displacement or offsetting of more carbon-intensive products or activities, which are typically considered in LCAs.^8^ To differentiate from conventional LCA methods, this paper refers to these carbon accounting techniques within MRV protocols as “CDR accounting”. Conventional land-based CDR, such as afforestation and reforestation, has more MRV protocols than emerging approaches such as biochar, enhanced rock weathering, or BECCS (Figure 1). The challenges in developing harmonized and robust MRV protocols have been discussed previously.^9–12^ The widespread use of LCA as a holistic environmental impact assessment tool holds significant potential to support MRV development.

**Figure 1:**
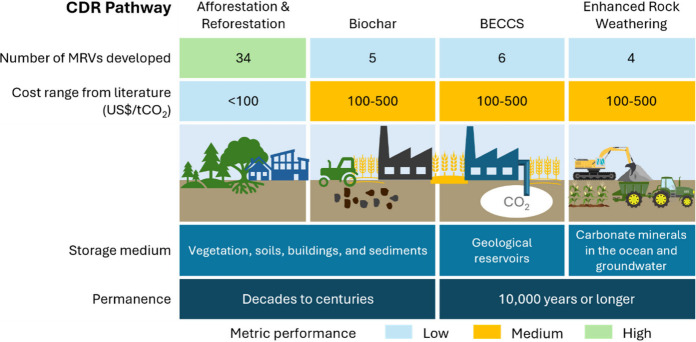
Four major CDR pathways in this perspective. The number of MRVs developed represents those published from 2003 to 2023.^2,17^ CDR cost ranges are collected from the literature.^17^ Adapted from ref 17. Copyright 2023 UNEP.

This Perspective aims to bridge the LCA and MRV communities in the context of land-based CDR, focusing on four methods: BECCS, biochar, enhanced rock weathering, and afforestation and reforestation. BECCS encompasses a variety of biochemical and thermochemical conversion technologies to turn biomass into various energy products while capturing and storing CO_2_.^13^ In recent years, BECCS has gained increased attention, including a shift from BECCS to a broad focus on biomass carbon removal and storage, referred to as “BiCRS” in the United States.^14^ Biochar, a carbon-rich material derived from biomass pyrolysis, can be used as a soil amendment for reducing GHG emissions and removing CO_2_.^15^ Enhanced rock weathering involves applying crushed silicate rocks to soil to facilitate CO_2_ removal.^16^ Afforestation and reforestation involve planting trees to increase the forest land cover. These methods represent both emerging and conventional CDR approaches. Figure 1 highlights their MRV progress, costs, storage medium, and permanence.

This Perspective focuses on the following questions: How have LCA concepts, methodologies, and data been utilized in the CDR accounting within MRV protocols in the voluntary carbon market? What are the similarities and differences between LCA and CDR accounting within MRV protocols for these four CDR approaches? How can LCA support the development of more robust MRV processes? What are the opportunities for future LCA research to enhance CDR assessment from an MRV perspective?

To answer these questions, we reviewed recent (within 5 years) or highly cited LCA studies. We compared the critical elements of GHG modeling and accounting methods in these LCA studies with MRV protocols from major carbon registries for the four CDR approaches in the voluntary carbon market. Tables 1 and 2 summarize these essential components, identified based on previous CDR literature.^2,4^ Detailed documentation of each study and protocol is provided in the Supporting Information.

**Table 1:** Summary of Essential Components of LCAs of Land-Based CDRs

	Afforestation and Reforestation	Biochar	BECCS^a^	Enhanced Rock Weathering
**# of studies**	6	26	28	5
**Functional Unit**	Areas of reforested land (5 studies); 1 metric ton (t) C sequestered or emissions reduced (1 study)	1 unit mass of dry feedstock (11 studies); 1 ha of managed land (5 studies); 1 unit mass of biochar (3 studies); bioenergy output (2 studies); household waste utilization (1 study); the combination of the above (3 studies)	1 kWh, MJ, or kg of energy product (18 studies); 1 unit dry mass of biomass or 1 ha of land (3 studies); 1 t carbon or CO_2_ removed (4 studies); others (3 studies)	1 t CO_2_ or CO_2_e removed or captured (4 studies); total cropland areas deployed (1 study)
**System Boundary**	Forest growth, operation and transport; 5 studies include soil organic carbon (SOC) change and timber extraction; wood products (2 studies); full life cycle (only 1 study)	Cradle-to-grave (24 studies); cradle-to-gate (2 studies)^a^. Feedstocks include: residue biomass (14 studies); urban or industrial waste (8 studies); energy crops (3 studies); both residues and energy crops (1 study)	Cradle-to-grave (16 studies) or cradle-to-gate (12 studies) with inconsistent definitions across studies	Cradle-to-grave but without runoff or leakage at enhanced rock weathering end of life (3 studies); cradle-to-gate (1 study); others (1 study)
**Impact indicators**	GWP-100. 1 study considered other impacts	GWP-100. 13 studies considered other environmental impacts	GWP-100. 12 studies considered other environmental impacts	GWP-100. 4 studies considered other environmental impacts
**Baseline (counterfactual scenario)**	3 studies considered baselines: conservation reforestation or unmanaged plantation	18 studies considered baselines: biomass left on the field or landfilled, or used for energy; natural forest regrowth (baseline for energy crops)	24 studies considered baselines: the same systems without CCS or fossil-based references with/without CCS	Not considered
**Timing**	All studies considered the timing	7 studies used time-dependent modeling of biochar decay	5 studies considered	Only 1 study specified 100 years
**Permanence**	2 studies considered wood product C permanence	20 studies considered using stable carbon ratio assumptions	4 studies considered CO_2_ losses	Permanence is not considered
**Multifunctionality**	Substitution/system expansion (2 studies)	System expansion (21 studies); energy allocation (1 study)	Substitution/system expansion (10 studies); energy allocation (2 studies)	Not considered
**Negative Emissions** ^b^	A (all studies); B (3 studies)	A (25 studies); B (22 studies); C (15 studies): reduce fertilizer and soil emissions, increase crop yield and SOC	A (all studies); B (12 studies); C (1 study): increased SOC	A (all studies)
**Direct Land Use Change**	All considered	3 studies considered; 15 studies used residues without considering SOC changes due to residue removal	6 studies considered	Not considered
**Indirect Land Use Change**	Not considered	1 study considered; 3 studies involve possible indirect land use change	2 studies considered	Not considered
**Uncertainty Sources and Methods**	Monte Carlo simulations (1 study); sensitivity analysis (2 studies)	Monte Carlo simulations (6 studies); sensitivity analysis (12 studies); both (3 studies)	Monte Carlo simulations (2 studies); sensitivity analysis (10 studies)	Monte Carlo simulations (2 studies); sensitivity analyses (3 studies)

a BECCS: bioenergy combined with carbon capture and storage.

b Cradle-to-grave and cradle-to-gate are two common system boundaries used in LCA. A cradle-to-grave system boundary typically encompasses raw material acquisition, production, transportation, use phase, and end-of-life stages. In contrast, a cradle-to-gate system boundary includes similar upstream activities but excludes the use phase and end-of-life stages.

c Negative emissions: A: Carbon removal. B: Avoided emissions due to material/energy substitution. C: Negative emissions due to co-benefits.

**Table 2:** Summary of Essential Components of MRV Protocols for Land-Based CDRs in the Voluntary Carbon Market

	Afforestation and Reforestation	Biochar	BECCS^a^	Enhanced Rock Weathering
**Protocols reviewed**	Gold Standard, Verra, American Carbon Registry, and Climate Action Reserve	Verra, Puro.earth, and Climate Action Reserve	Puro.earth, American Carbon Registry, and Gold Standard	Puro.earth and Isometric
**Functional unit**	1 project area or 1 project per year or over a time period	1 project/year or project over a time period	1 project/year or project over a time period	1 project over a time period
**System boundary**	Carbon pools differ by protocol but do not include life cycle activities	Cradle-to-grave	Cradle-to-grave for CO_2_, but differ in infrastructure, materials, and fuels	Cradle-to-grave but differ in infrastructure activities
**Impact indicators**	Net t of CO_2_e removal without specifying GWP methods	Net t of CO_2_e removal, using GWP-100	Net t of CO_2_e removal, using GWP-100	Net t of CO_2_e removal, using GWP-100
**GHG types**	Mainly CO_2_, other GHG inclusions differ by protocol	2^a^ specify GHG types, but not system boundaries of GHG factors	2 specify GHG types. The system boundaries of GHG emission factors vary by protocol	All specify GHG types. One requires LCA results grouped by life cycle stages and GHG
**Baseline for additionality**	Baseline carbon stock inclusion and methods differ by protocol	Zero emissions as default. Non-zero emissions are allowed in 2 protocols	2 include baselines and consider situations without a CCS project	Baseline: non-enhanced rock weathering application
**Additionality**	Regulatory, carbon performance, and financial additionality	Regulatory, performance, and financial additionality differ by feedstock	Regulatory, performance, and financial additionality	Regulatory, environmental, and financial additionality
**Timing**	100 years or a crediting period	100 years	Vary by protocol, including 10 years, 40 years, or more.	100 years for GWP, but weathering can be longer
**Permanence**	2 considered	Considered by parametrized estimation	Considered and require demonstration and monitoring	Considered and require measurement and simulations
**Multifunctionality**	Not mentioned	2 use energy allocation	1 suggests using LCA standards	Depends on protocol
**Negative Emissions** ^b^	A	A and B	A	A
**Direct Land Use Change (DLUC)**	Carbon stock change between baseline and project reflects DLUC	Limit DLUC by requiring no ecosystem carbon losses	1 refers to RED II sustainability criteria, 1 includes SOC losses due to land clearing	1 includes, and the other includes when DLUC change leads to increased emissions.
**Indirect Land Use Change (ILUC) and other indirect impacts**	Methods and assumptions differ by protocol	Limit IDUL by excluding dedicated biomass. One allows purpose-grown biomass from marginal lands or reclaimed mining sites	Not mentioned	Both standards ask for leakage emissions.
**Use of LCA methodology and/or data**	Not mentioned	1 uses LCA principles and standards. One uses the life cycle concept	2 use LCA methods and LCA tools and data	Use and be informed by LCA principles and standards
**Uncertainty**	3 considered	1 includes QA/QC for main data and parameters. One specifies confidence intervals for sampled and lab data	All considered. Two identified uncertainty sources and QA/QC requirement	1 offers a checklist of uncertainties, evaluation, and validation. One requires reporting uncertainties and sensitivity analysis

a BECCS: bioenergy combined with carbon capture and storage.

b For conciseness, the table lists the number of protocols. The Supporting Information Excel file provides detailed information on specific protocols and their methods.

c Negative emissions: A: Carbon removal. B: Avoided emissions due to material/energy substitution. C: Negative emissions due to co-benefits.

Based on Tables 1 and 2, we have summarized the main similarities, differences, and gaps between LCA and CDR accounting within MRV protocols in Figure 2. We identified gaps in MRV protocols where LCA can provide valuable support, including baseline emission estimation, uncertainty analysis, environmental safeguards, and data for more comprehensive GHG emission factors and overlooked carbon pools.

**Figure 2:**
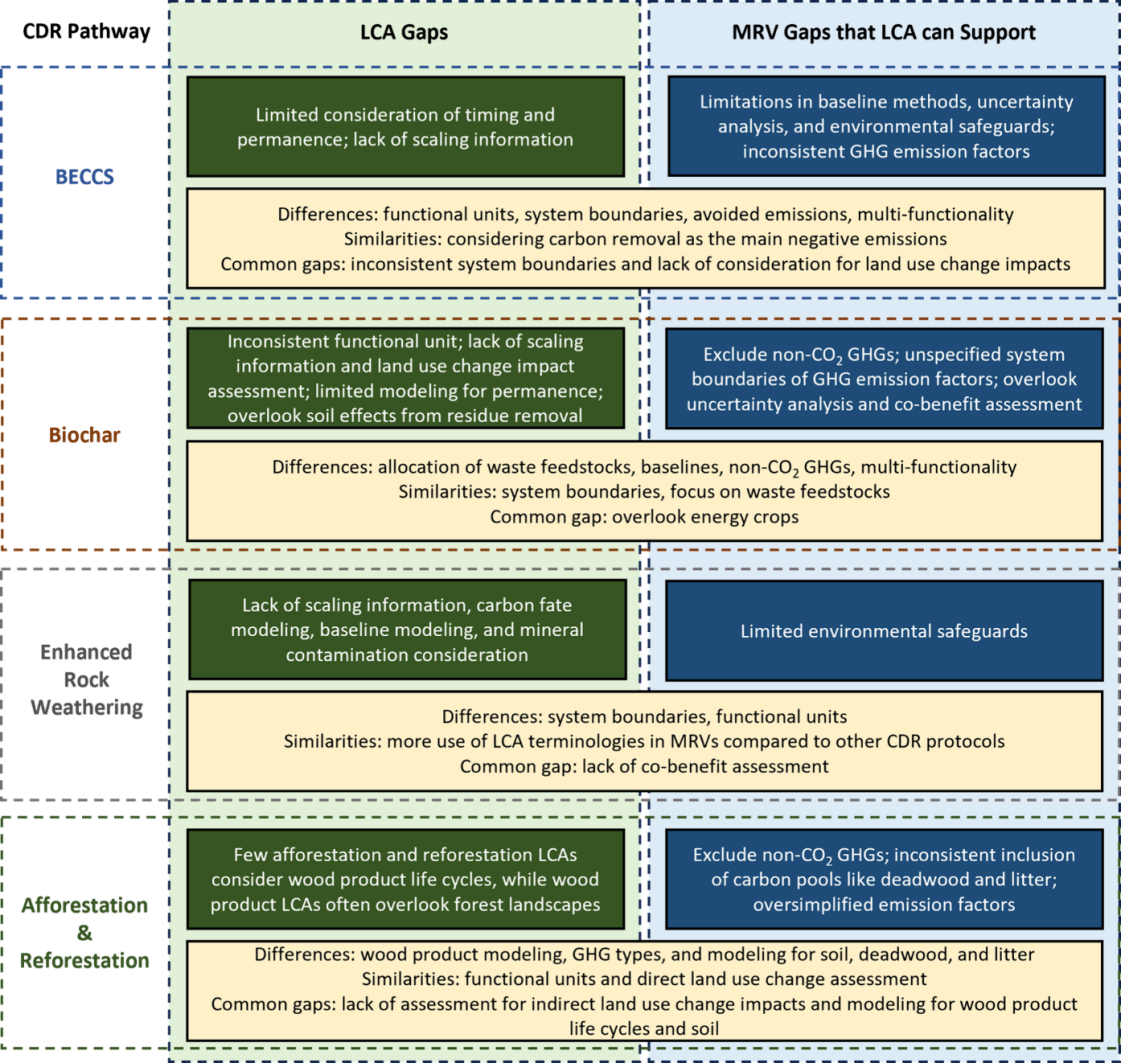
Summary of the main similarities, differences, and gaps between LCA and CDR accounting within the MRV protocols.

Our analysis also identified several LCA gaps crucial for large-scale CDR assessment, such as the limited consideration of timing and permanence as well as the challenges in scaling LCA results to project-level CDR accounting. Future LCA research should address these issues and focus on aspects vital to the CDR assessment. Some CDR methods bring co-benefits, such as improved crop yields from biochar and enhanced weathering. Currently, the assessment of co-benefits is a gap in both LCAs and MRV protocols. Future LCAs should concentrate on two key questions: (1) How significant are these co-benefits in the context of life cycle GHG balances and various environmental impacts? (2) What are the most robust methodologies to incorporate these co-benefits into comprehensive CDR project assessments? Another common gap in LCAs and MRV protocols is the lack of land use change impact assessment. We recommend leveraging advanced modeling tools, such as integrated assessment models, tailored to each CDR method to address these issues.

The findings for each CDR method are detailed in the following sections along with suggestions for future LCA research to support the development of robust MRV protocols and the assessment of CDR projects.

## Bioenergy Combined with Carbon Capture and Storage (BECCS)

While MRV protocols for BECCS utilize LCA methods, they differ significantly in their functional unit and system boundaries. LCAs typically focus on the product or process level using varied functional units, such as the mass or energy content of products (Table 1), which differ greatly from the project-level CDR accounting^18^ used in MRV protocols. This difference complicates direct comparisons between the LCA results and project-level carbon credits. For instance, previous LCAs show a large range from −3048 to 1750 gCO_2_e/kWh for BECCS producing electricity,^6,19–28^ from −35 to −159 gCO_2_e/MJ for BECCS producing biofuels,^29–34^ and from −8 to −200 kgCO_2_e/kg H_2_ for BECCS producing H_2_.^6,13^ This difference also challenges the direct comparison between the LCA results and the carbon credits reported for a BECCS project. Aligning LCA results with project-level CDR accounting metrics (e.g., annual project CO_2_ removal, Table 2) requires additional data that are often missing in LCA studies, e.g., biorefinery annual production, capacity, and lifespan. Furthermore, LCAs commonly use cradle-to-gate system boundaries focused on energy products, while MRV protocols use cradle-to-grave boundaries based on the CO_2_ life cycles. For example, most cradle-to-gate LCAs of hydrogen and biofuels cover biomass supply chains, energy production, and CCS but exclude fuel distribution and end-use.^13,27,30,33,35–40^ In contrast, MRV protocols and some LCA studies include similar activities but define a cradle-to-grave boundary based on CO_2_ life cycles,^13,26,28,34,41^ which are different from other cradle-to-grave LCAs of biofuel covering biofuel end-use.^29,31,32,42^ This difference can cause confusion, especially when similar activities are defined differently. Future BECCS LCAs and MRV protocols should clearly define boundaries and provide transparent process diagrams to avoid misunderstandings.

MRV protocols and LCAs are consistent in treating carbon removal as the primary source of negative emissions, but LCAs also account for avoided emissions from substitutions (e.g., replacement of fossil fuels). This substitution is relevant to the multifunctionality issue, a challenge in CCS carbon accounting. In LCA, CCS captures and stores CO_2_, transforming it from an elementary flow into a product flow, creating ambiguity in assessing the environmental impact of energy products and CO_2_.^43^ Some LCA studies treat CO_2_ and energy products as the main products and use methods like system expansion, while others only include energy outputs as the main products and use energy allocation (Table 1). One study examined different approaches and recommended the substitution method, which is mathematically equivalent to system expansion, to determine the carbon footprint of captured CO_2_.^43^ The study also recognizes the possible negative results, given that the substituted/avoided systems are often carbon-intensive.^43^ These negative results should be interpreted as avoided emissions rather than CDR. It is also critical for LCA studies to transparently provide detailed breakdowns of the results. A recently proposed CDR accounting framework highlights the importance of distinguishing between carbon removal and offsetting.^8^ Effective CDR must achieve net removal of CO_2_ from the atmosphere, whereas avoided emissions result from substituting or offsetting more carbon-intensive alternatives. MRV protocols do not explicitly address this issue, but one protocol excludes non-carbon removal activities to focus on CCS parts. As MRV generally focuses on net CDR rather than system-wide effects, extra attention is needed when CDR projects use data from LCA studies that show net negative results.

Most LCAs model product substitution by choosing a traditional product/product mix and deducting average life cycle GHG emissions based on the substitution ratio. This approach has been criticized in consequential LCA studies that show differences between marginal and average suppliers. For instance, one study^44^ shows small changes in oil demand (−2.5%) lead to the displacement of crude with 25–54% higher GHG emissions intensity than the global average. When assessing CDR projects, LCA incorporating avoided emissions must carefully identify marginal suppliers and counterfactuals and transparently document their emission data. It is essential to conduct uncertainty and sensitivity analyses if it is necessary to use average data in an LCA for CDR. These analyses can help understand how variability in substitution affects the overall GHG mitigation impacts.

The LCA community can support future MRV protocols for BECCS by offering insights into baseline comparisons and uncertainty. Most LCAs include baselines, comparing systems with and without CCS or against fossil-based references. While most MRV protocols also consider baselines, only the American Carbon Registry protocol^45^ specifies methods, including a project-based baseline (a counterfactual scenario without CO_2_ capture) or a standard-based baseline that uses similar or different technology to fulfill the same purpose and function.^45^ The lack of consistent, specified methods for establishing and estimating baseline emissions is a critical challenge for CDR MRV.^46^ LCA studies with transparent inventory data for both baselines and BECCS can offer valuable data and insights into calculating baseline emissions in MRV protocols that include baselines but lack specified data sources and methods. LCAs also provide valuable examples of addressing uncertainty through sensitivity analysis or Monte Carlo simulations. While all MRV protocols acknowledge uncertainty, they mainly focus on identifying sources and applying Quality Assurance/Quality Control (QA/QC) procedures. Uncertainty analyses in LCA studies can help identify critical uncertainty sources and prioritize mitigation strategies.

LCA provides valuable data sets for GHG emission factors and environmental safeguards, filling gaps in BECCS MRV protocols. Inconsistent GHG types and system boundaries for emission factors across protocols can lead to varied results. For example, GHG emission factors in the American Carbon Registry protocol^45^ only include fuel combustion and electricity generation. While the Gold Standard protocol^47^ uses GHG emission factors considering the full life cycles using LCA tools such as GREET.^48^ This discrepancy can cause significant variations. For instance, renewable energy might appear as zero-emissions when considering only electricity generation, but their entire life cycle often shows non-zero emissions, such as 98.3 to 149.3 gCO_2_e/kWh for utility-scale solar.^49^ Previous literature also showed higher GHG emissions for the regional-average electricity grid when considering the entire life cycle (615 gCO_2_e/kWh) compared to only the electricity generation phase (470 gCO_2_e/kWh).^50^ Leveraging LCA tools and databases can provide more thorough and accurate GHG emission factors for the energy and materials used in CDR projects. To balance comprehensiveness with practicality, we recommend using full lifecycle GHG emission factors when available, with clear justifications for any exclusions. Some LCA studies have quantified environmental impacts beyond climate change, such as human health and resource availability,^51^ offering insights for developing environmental safeguards—an area under-addressed in current MRV protocols, but environmental concerns on BECCS have been widely discussed in the literature.^52^

Future LCA should consider timing and permanence, crucial aspects overlooked in most LCAs but intensively discussed in MRV protocols (Tables 1 and 2). Dynamic LCAs have pinpointed important temporal dynamics related to BECCS, including land use changes,^5^ soil organic carbon,^37^ future decarbonization of energy systems and supply chains, and time-dependent climate impacts of GHG emitted at different time.^26^ These studies leverage dynamic modeling methods, including dynamic LCA, ecosystem modeling, and integrated assessment models. Future LCAs and CRD accounting in MRV protocols should consider these advanced modeling tools, especially for the impact of land use change. Only the Gold Standard protocol^47^ has considered direct land use changes from project infrastructure, while others rely on sustainable biomass criteria like RED II^53^ without quantitative assessment. In addition, future LCAs should consider carbon leakage in storage sites, a significant gap in previous studies and a critical factor for ensuring permanence, leveraging recent advancements in geological dynamic approaches.^54,55^

## Biochar

Biochar MRV protocols use biomass feedstocks and cradle-to-grave system boundaries similar to those of LCA studies, covering raw material acquisition, biomass transportation, biochar production, distribution, and end-use. All three MRV protocols specify eligible biomass feedstocks. The Verra protocol restricts feedstock to waste biomass, including forest and agricultural residues and industrial and urban wastes.^56^ Puro.earth includes sustainably sourced biomass^57^ like those on the positive list of the European Biochar Certificate.^58^ This focus on waste biomass aligns with that of biochar LCAs. However, MRV protocols often assume waste biomass is burden-free, while some LCAs allocate environmental burdens using mass or economic allocation.^59–63^ This raises questions about how to attribute burdens when waste materials gain value as feedstock. Different approaches may be needed over time, given changes in the market and counterfactuals.^64^ Energy crops are largely overlooked. Only one protocol (Climate Action Reserve^65^) includes purposely grown biomass from marginal lands or reclaimed mining sites, excluding commodity crops within 3 years of the project and requiring no ecosystem carbon loss. Future LCAs could explore these criteria to assess the environmental implications of using energy crops for biochar, particularly as growing interest in co-producing biochar and biofuels from energy crops on marginal lands.

CDR accounting in biochar MRV protocols differs from biochar LCA in addressing baseline and multifunctionality. MRV protocols often set a zero-emission baseline, although some allow non-zero baselines, e.g., the Verra protocol, but it considers only CO_2_, excluding other GHGs.^56^ In contrast, biochar LCAs model counterfactual scenarios and include non-CO_2_ GHGs, e.g., CH_4_ from landfilling sewage sludge.^66–71^ These LCA methods and data can support future MRV protocols for estimating the emissions of non-zero-emission baselines when needed. Another difference is in multifunctionality. MRV protocols generally use energy allocation, while LCAs prefer system expansion (Tables 1 and 2). ISO 14044 ^72^ recommends a stepwise procedure, noting that system expansion can reflect the economic and physical implications of coproducts but requires more data, and different modeling choices can result in low transparency and high variability.^73^ Allocation based on physical relationships is simpler and more reliable in terms of data availability, but it may not accurately reflect the drivers or intention of industrial processes,^73^ e.g., energy allocation may be unsuitable when biochar is not used as an energy product. Sensitivity analyses for different allocation methods with transparent documentation can help clarify results and quantify uncertainties from methodological choices.

Biochar LCAs can support MRV protocols by providing insights into uncertainty and GHG emission scopes. Two protocols have addressed uncertainty issues by specifying QA/QC procedures or confidence intervals for some data and parameters critical to the permanence, which is a significant source of uncertainty. However, these protocols do not cover many other uncertainty sources identified by biochar LCAs, such as GHG emission factors associated with biochar production and energy use. Most biochar LCAs have used Monte Carlo simulations to quantify the variations of net CO_2_e removal potential and sensitivity analyses to identify the main driving factors. Their results can help prioritize QA/QC procedures for data with large uncertainty. In addition, biochar protocols reviewed do not specify system boundaries of GHG emission factors; one does not specify the types of GHGs included. Future MRV protocols should address this issue to ensure a consistent scope of all of the GHGs included. Biochar LCAs can provide holistic GHG emission factors and identify significant GHG emission sources.

In addition, biochar LCAs have explored various co-benefits and their significance, an overlooked aspect in biochar MRV protocols. These co-benefits include decreased fertilizer use,^66,68–71,74–78^ reduced N_2_O and CH_4_ emissions,^61,63,66,68,69,74,76–81^ increased crop yield,^77–79,81^ and enhanced SOC stock^61,75,77,80,82^ (see Table 1, negative emissions). The co-benefits of biochar could be the main drivers of its adoption in the agriculture sector.^83,84^ The importance of assessing CDR co-benefits has been increasingly recognized by stakeholders and policymakers. For instance, the European Union Certification Framework of Carbon Removals requires that “Carbon removal activities must have a neutral impact on, or generate a cobenefit for other environmental objectives.”^85^ Studies have found that projects with co-benefits can attract higher price premiums,^86–88^ and highlighted the need to understand the significance and develop co-benefit assessment methods for future MRVs.^2^ Biochar LCAs offer valuable data sets and quantitative insights into the significance of various co-benefits in life cycle GHG balances and other environmental impacts across different use cases. These insights can assist the MRV community in identifying priority areas and directing future efforts. Future LCA research should explore robust, verifiable co-benefits assessment methods to support MRV protocol development.

This comparison between biochar MRV protocols and LCAs pinpoints opportunities to improve future LCAs. First, using a consistent functional unit across studies, such as biochar applied to a designated area over a specific time period, would significantly enhance the comparability between LCA studies and carbon credits reported for a biochar project. Previous LCA studies have used varied functional units and reported results ranging from −1.1 to −9.9 kgCO_2_e/kg biochar^62,81,89^ and from −0.5 to −47.8 tCO_2_e/ha.^78,79,90,91^ Some studies report a wider result range from negative to positive (from −2,800 to 1,355 kgCO_2_e/t feedstock processed).^34,59,63,66,70,71,74–76,92^ These positive results (net GHG emitting) are predominantly associated with high-moisture feedstocks such as sewage sludge, which require substantial energy input for drying. Comparing these LCA results and scaling them to the project-level CDR accounting requires additional information such as feedstock types, biochar yield, production capacity, application rates, and areas. Not all biochar LCAs provide such information. Future LCA studies should use consistent functional units and transparently disclose information for scaling. Second, biochar LCAs should integrate dynamic modeling for carbon permanence. While MRV protocols use consistent 100-year parametric modeling from Woolf et al. 2021,^93^ LCA studies use simpler assumptions^61,62,66,77,78,91,92,94^ or different decay models.^60,68^ Future biochar LCAs should incorporate advanced models from recent research^93^ and the latest data from biochar meta-analyses.^95–97^

Biochar LCAs need better modeling of the land use change impact. Few studies consider direct or indirect land use change. Many studies using agricultural and forest residues overlook SOC effects. Recent LCA shows SOC loss from forest residual removal as a significant GHG source, even when 50% of residues are left on the land.^98^ Long-term SOC loss can reduce biomass productivity, affecting the resilience of biomass supply chains. Future LCAs should consider these dynamics and be careful about the assumption that residue biomass is carbon neutral or environmentally burden-free. For indirect land use changes, biochar LCAs can benefit from enormous modeling efforts in biofuel LCAs.^99^ Many biofuel LCAs have used integrated assessment models such as GTAP,^100^ GLOBIOME,^101^ and GCAM^102^ to simulate the indirect land use change impact. Biochar LCA can use similar approaches, such as Bergero et al.^103^ who modeled biochar in GCAM. Incorporating biochar into integrated assessment models and developing indirect land use change GHG emission factors, as those for biofuels,^104^ will enhance LCA and MRV for biochar projects.

## Enhanced Rock Weathering

Enhanced rock weathering is a newer CDR, with only five LCA studies and two MRV protocols reviewed. Compared to other CDR methods, the two MRV protocols of enhanced rock weathering incorporate more LCA terminologies, but there are still differences in the system boundary and functional unit. For example, both MRV protocols use cradle-to-grave system boundaries, but one includes the construction or manufacturing of infrastructure. These activities are usually omitted in LCAs. Including these activities in future LCAs could clarify their importance for MRV. Another difference is the functional unit. Most LCAs have used 1 unit mass of CO_2_ or CO_2_e removed or captured, reporting their embodied GHG emission range from 41 to 359 kgCO_2_e/tCO_2_ captured or removed;^16,50,105,106^ one study reports embodied GHG emissions of 842 kgCO_2_e/ha at an application rate of 50 t/ha.^105^ While MRV protocols use 1 project as their basis, with additional details required by the Puro.earth protocol,^107^ such as application rate, material type, and areas. Some LCAs report application rates and areas,^50,105,106^ while others do not.^16,108^ Future LCAs should include these data for scalability.

Compared to MRV protocols, previous enhanced rock weathering LCAs have several gaps, particularly in accounting for carbon fate, baselines, and mineral contamination. Carbon fate is the “end of life” for carbon captured by enhanced rock weathering in dissolved weathering materials. This is mentioned in MRV protocols and should be included in the cradle-to-grave system boundary. However, previous cradle-to-grave LCAs do not consider carbon fate,^16,106,108^ and one LCA uses a cradle-to-gate boundary, excluding downstream impacts like carbon runoff or leakage.^50^ This is also related to the permanence and timing, two aspects overlooked in previous LCAs. Another gap is the baseline. Both MRV protocols set the baseline as no enhanced rock weathering application, either as a common agricultural practice or as natural weathering. None of the LCAs consider these baselines; including these counterfactual scenarios in LCAs would provide a holistic assessment. In addition, while all LCA studies evaluate environmental impacts beyond climate change, only one considers potential mineral contamination (e.g., metals).^106^ Including this in future LCAs would help to develop environmental safeguards for enhanced rock weathering projects.

Both MRV protocols mentioned co-benefits reported in the literature, such as decreased N_2_O emissions,^109,110^ improved crop yields, and reduced fertilizers.^109,111–114^ However, due to the lack of standardization, they do not include quantitative assessments of these co-benefits. None of the enhanced rock weathering LCA studies have included these co-benefits either. Assessing and incorporating co-benefits and potential risks like increased direct land use change emissions in future LCA would enhance our understanding of full life cycle impacts and support co-benefit assessment for CDR projects.

Addressing these gaps is crucial but challenging for LCA researchers and practitioners to tackle alone. Assessing carbon fate and co-benefits requires data on material runoff, land emissions, fertilizer usage, and crop yields before and after enhanced rock weathering applications. Obtaining these data is difficult due to complex chemical reactions, slow mineral weathering, and ecosystem variability—challenges also affect the MRV of enhanced rock weathering.^115^ As a result, MRV protocols allow for simulations combined with ongoing field measurements. Recent research has started to quantify soil and crop responses to mineral application, such as reduced soil N_2_O emission, improved crop yields, improved nitrogen use efficiency, and reduced phosphorus and potassium fertilization.^114^ However, these data are highly region-specific. As the field evolves, closer collaboration between the LCA and enhanced rock weathering communities will be essential for advancing and supporting robust MRV development.

## Afforestation and Reforestation

Compared to other CDR methods, afforestation and reforestation, and improved forest management, are more established, with more protocols published.^2^ Most CDR investments before 2021 were directed toward forestry startups, with stable growth since then.^2^ This perspective focuses on afforestation and reforestation, excluding improved forest management, to align with the identified LCA focuses. In total, we reviewed 4 MRV protocols and 6 LCA studies.

While most protocols do not explicitly mention LCA methods, the accounting approaches are similar in functional units and direct land use change assessment. Nearly all LCA studies used the forested area as the functional unit and reported timing over one or multiple rotation cycles, which are consistent with MRV protocols. Previous LCA studies reported average annual net carbon removal rates ranging from 0.4 to 5.4 tC/ha/year (defined as the total amount of net carbon removed during the simulation period divided by the number of years) depending on regions, time frame, and forest management strategies. These LCA studies provide valuable references for CDR projects in similar regions.^116–121^ Detailed regional specifics and LCA results are provided in the Supporting Information. Both protocols and LCA studies have considered direct land use changes by estimating net GHG flux changes on forested and non-forested lands, though different approaches are used for measuring SOC changes, e.g., the Gold Standard protocol defaults a rate of 1.8 tCO_2_/ha/year^122^ while Verra requires SOC when soil disturbance occurs.^123^

MRV protocols vary in their approach to wood product modeling, both among themselves and compared with LCA studies. Differences include the treatment of wood products in the system boundaries. The Gold Standard^122^ and Verra^123^ protocols exclude harvested wood products, while the American Carbon Registry^124^ and Climate Action Reserve^125^ include them. The American Carbon Registry protocol uses U.S.-specific and generic methods to estimate carbon retention in wood products, while the Climate Action Reserve protocol accounts for CO_2_ emissions from wood decomposition but excludes CH_4_ emissions, assuming future landfill control. None of these protocols consider the full life cycle GHG emissions of forest products. Among LCA studies, only two address wood products,^116,121^ with only one covering the full cradle-to-grave life cycle.^121^ This highlights the gap in both LCAs and MRV protocols. Many LCAs focus on wood products at the process or product level (e.g., 1 m^3^ of wood product^126^) without considering forest landscapes, limiting their relevance to afforestation and reforestation projects. Zhang et al. proposed a multi-scale LCA framework that integrates process-based LCA with landscape-wide modeling for afforestation and reforestation, offering a more comprehensive method for incorporating wood product life cycles and their emissions into forest GHG modeling and accounting.^121^ Future LCAs and MRV protocols are encouraged to consider this method.

Afforestation and reforestation protocols and LCA also differ in GHG types and modeling for soil, deadwood, and litter. Most MRV protocols focus solely on CO_2_ emissions, excluding other GHGs. While LCA studies include other GHGs like CH_4_ and N_2_O.^117,118,120,121^ Deadwood and litter are excluded from the Gold Standard^122^ and Climate Action Reserve protocols,^125^ but are optional in Verra^123^ and American Carbon Registry.^124^ In contrast, LCAs often include deadwood and litter,^116–121^ recognizing their potential for bioenergy or biochar production, which could enhance CDR when combined with afforestation and reforestation or BECCS. The previous multi-scale LCA study^121^ shows the greater GHG mitigation potential of combining afforestation and reforestation with emerging wood products like cross-laminated timber and biochar. Considering deadwood and litter as carbon pools can enable future projects for synergistic CDR solutions. In addition, LCAs offer more holistic emission factors for various GHG emission sources. For example, MRV protocols oversimplify fertilizer emissions: the Gold Standard protocol assumes 0.005 tCO_2_ per kg of nitrogen fertilizer,^122^ the Verra protocol only includes N_2_O emissions from fertilizer applications,^123^ while others omit fertilizers.^124,125^ The life cycle GHG emissions of fertilizers have large variations, and upstream production can make a large contribution.^127–130^ Previous studies have identified fertilizers as a significant source of GHG emissions in forest management activities.^119^ Neglecting these variations may result in under estimations of GHG emissions.

Indirect land use change is a key gap in both the LCA and MRV protocols. While MRV protocols address this as “activity shifting” by applying carbon stock change ratios to account for leakage caused by displaced commodities or activities, they use varying ratios and assumptions (see Supporting Information for details). None of the LCAs reviewed here have addressed indirect land use change, though it is an active research area in LCA for biofuels.^99^ Previous analyses have used LCA methods and global land-use modeling to assess the impact of future wood demand on land use change and GHG emissions on a global scale.^131^ Future research should explore how to downscale these findings for region-specific projects. One approach could be developing product-based indirect land use change GHG emission factors, similar to those for fuels, downscaled to a unit like 1 MJ of fuels.^131^ Alternatively, land use modeling and LCA studies could operationalize their findings based on 1 ha of land, facilitating their application in afforestation and reforestation projects, carbon markets, and policy development.

## Recommendations for Future Research

In this Perspective, we compared GHG accounting and environmental impact assessment methods between LCAs and MRV protocols across four major CDR methods. Our analysis highlights several key insights and opportunities for improvement.

Most MRV protocols use LCA concepts, methodologies, or data. LCA community can support future MRV protocols by providing critical insights into estimating baseline emissions and assessing additionality. LCA can also offer comprehensive data on often overlooked carbon pools, such as wood products, and provide insights into how effectively different methods address multifunctionality challenges. Uncertainty analysis and co-benefit considerations, integral to LCA, can support the development of more robust MRV protocols. LCA literature can be reference points for CDR projects to compare and validate their GHG modeling and carbon credit estimation. However, it is important to be mindful of potential differences in system boundaries, functional units, methodological choices, and temporal and geospatial scopes. As the objectives of LCA and MRV differ, we do not intend to recommend that MRV protocols should always adopt the LCA best practices. Instead, our analysis demonstrates how the LCA community can support MRV protocols with valuable datasets and lessons learned.

Among the four CDR pathways, biochar and BECCS have the most LCA publications, providing valuable references for these CDR projects. In contrast, enhanced rock weathering remains in its early stages; more LCAs are needed to support future projects. Although LCA publications for afforestation and reforestation are limited, the wealth of studies on wood products can support future afforestation and reforestation projects that consider wood products.

Future CDR LCA research should prioritize issues around timing, permanence, scaling, and negative emissions. Dynamic modeling approaches are essential for accurately capturing GHG flows with known temporal patterns, such as biochar decay, forest carbon sequestration, and SOC changes. It is crucial to address the permanence, which is often overlooked in current LCAs. The LCA community should leverage recent advancements in dynamic and parametric modeling for CDR techniques. Furthermore, transparent documentation of scaling information is vital to support project-level CDR accounting. Some LCA studies have considered negative emissions beyond CO_2_ removal, such as product substitutions and co-benefits. Clear documentation of data sources, disaggregated results, and assumptions, such as substitution rates and co-benefits data, is crucial to avoid misleading conclusions and potentially overestimating net GHG mitigation potential.

LCA studies can vary significantly in their functional units, system boundaries, data sources, and modeling methods. LCA methods used in academic research may not always align with the practical requirements of policy or industrial applications. Harmonization studies in areas like electricity generation^132,133^ and aviation fuels^134^ provide insights into the impact of data and modeling uncertainties, offering guidance for improving robustness and standardization in LCA applications within industry and policy areas, e.g., low-carbon fuel standards.^99^ With more CDR LCAs being published, harmonization and meta-analysis are needed to identify key areas for improvement. This will enable the development of reliable LCA applications that can support MRV protocols, while recognizing the distinct goals and practical considerations of each approach. Land use change remains a critical gap in both the LCA and MRV protocols. While some MRV protocols attempt to minimize carbon leakage from direct land use change by limiting biomass sourcing to residues, recent LCAs indicate significant GHG emissions from SOC changes due to agriculture and forest residue removal.^98,135,136^ Future LCAs and MRV protocols should consider soil impacts rather than assume that residue biomass is always carbon neutral. Assessing indirect land use change is challenging with methods varying across MRV protocols. There is ongoing active land use change research, mostly for biofuels. We recommend adopting similar approaches, such as integrated assessment models, to develop GHG emission factors for indirect land use change applicable to both LCA and project-level CDR accounting.

Foresting collaborations between the LCA and CDR communities to develop robust MRV protocols will benefit both communities. Future LCAs should align with MRV processes, while MRV protocols can benefit from data, state-of-the-art tools, and knowledge in LCA. While this perspective focuses on four main CDR methods, emerging approaches, such as biomass burial^137^ and ocean alkalinity enhancement, present significant opportunities. Although ongoing efforts^138^ are being made, these emerging approaches have much fewer established LCAs and MRV protocols than CDR methods discussed in this paper, presenting excellent opportunities for future collaborative projects and case studies.

## Supplementary Material



## Notes

Detailed information on MRV protocols reviewed for afforestation and reforestation (sheet “AR Protocol”), BECCS (sheet “BECCS Protocol”), biochar (sheet “Biochar Protocol”), and enhanced rock weathering (sheet “ERW Protocol”); Detailed information on LCA studies reviewed for afforestation and reforestation (sheet “AR LCA”), BECCS (sheet “BECCS LCA”), biochar (sheet “Biochar LCA”), and enhanced rock weathering (sheet “ERW LCA”) (XLSX)
